# Longitudinal associations between relationship quality and prosocial behavior in Chinese adolescents: the mediating role of meaning in life

**DOI:** 10.3389/fpsyt.2026.1915264

**Published:** 2026-09-11

**Authors:** Li Meng, Chengxi Li, Tianqinq Dai, Shaojun Zhang, Bi Zhang, Qin Yang

**Affiliations:** 1 School of Educational Science, Hunan Normal University, Changsha, Hunan, China; 2 Changjun Bilingual School, Changsha, Hunan, China; 3 Changsha Preschool Education College, Changsha, China; 4 Research Center for Rural Education, Hunan Normal University, Changsha, Hunan, China; 5 Hunan University of Chinese Medicine, Changsha, Hunan, China; 6 School of Preschool Education, Changsha Normal College, Changsha, Hunan, China

**Keywords:** friendship, longitudinal mediation model, meaning in life, parent-child relationship, prosocial behavior, teacher-student relationship

## Abstract

**Objective:**

Positive youth development (PYD) can be enhanced through dynamic interactions between individuals and their environments. While previous research has primarily focused on its social-emotional learning mechanisms, the cognitive and motivational pathways of PYD were seldom explored. To address this gap, this study investigated the associations of parent -child relationships, teacher–student relationships, and friendships with adolescents' prosocial behavior, with a focus on the mediating role of meaning in life.

**Methods:**

A three-wave temporally ordered mediation analysis was conducted using three waves of data collected at six-month intervals from 671 Chinese junior high school students.

**Results:**

The findings revealed that the quality of parent–child relationships and friendships at Wave 1 was positively associated with both the presence and search for meaning in life at Wave 2, with friendships showing stronger associations, whereas teacher–student relationships were not significantly associated with either dimension of meaning in life. Furthermore, only the presence of meaning in life at Wave 2 was positively associated with prosocial behavior at Wave 3, while the search for meaning in life was not significantly associated with prosocial behavior.

**Conclusion:**

These findings provide practical insights into China's moral education framework, rooted in Confucian doctrine, by suggesting that high-quality relationships are associated with adolescents' sense of societal contribution as an aspect of life's meaning. Possible reasons for the nonsignificant roles of teacher–student relationships and the search for meaning in life were discussed, along with the study's limitations and directions for future research.

## 1 Introduction

Prosocial behavior is defined as voluntary actions aimed at benefiting others, such as helping, sharing, and comforting (1). A growing body of scientific research suggests that prosocial behavior can predict various positive developmental outcomes, including well-being (e.g., 2) and academic success (e.g., 3). Therefore, gaining a deeper understanding of the factors that promote prosocial behavior is crucial for fostering the positive development of adolescents.

The positive youth development (PYD) framework asserts that all youth possess the potential for growth and development, which can be optimized through the dynamic interactions between individuals and their environments (4). Prosocial behavior, as a component of PYD, arises from reciprocal relationships between individuals and their social contexts (e.g., parent-child relationships, teacher-student relationships, and friendships), which contribute to the well-being of both parties (5). Extending the PYD perspective to the Chinese cultural context, the present study considers prosocial behavior through the lens of Confucian ethics, particularly the principle of benevolence, which emphasizes compassion, interpersonal care, and moral responsibility toward others (6).

A great number of studies have examined the underlying mechanism of this process through the lens of social-emotional learning (e.g., 7). Specifically, high-quality relationships can foster adolescents’ prosocial behavior in two primary ways: by serving as models of prosocial behavior and by providing a warm, supportive relational context (8). According to social learning theory, adolescents acquire prosocial behaviors by observing and imitating the actions of others, particularly within close relationships (9). Furthermore, strong, supportive relationships with family members and peers can enhance the internalization of prosocial values and behaviors (10). When adolescents experience secure attachments with family and friends and feel accepted and supported, they are better equipped to recognize others’ needs and exhibit prosocial behaviors (11).

However, relatively few studies have examined this process from cognitive and motivational perspectives. Theories of reasoned action and planned behavior suggest that attitudes and behavioral intentions guide behavior (12–14). Cognitive dissonance theory further suggests that individuals are motivated to reduce inconsistencies among their beliefs, values, and actions (15, 16). Meaning in life reflects the extent to which individuals experience their lives as coherent, purposeful, and significant (17). Meaning-maintenance and meaning-making models propose that broader beliefs and goals help individuals interpret experience and maintain coherence (18, 19). When prosocial values are integrated into adolescents’ life goals, helping others may represent behavior that is consistent with those goals. Consistent with this reasoning, the presence of meaning has been positively associated with prosocial behavior (20), and prosocial behavior may, in turn, strengthen meaning in life (21–23).

Social relationships are an important source of meaning in life. Supportive relationships with parents, teachers, and peers may provide adolescents with a sense of belonging and connectedness, which are associated with greater meaning in life (24, 25). Meaning in life may, in turn, orient adolescents toward behaviors that are consistent with their values and goals, including prosocial behavior. Accordingly, the present study examines whether the presence of meaning in life and the search for meaning in life serve as potential mediating pathways linking parent–child relationships, teacher–student relationships, and friendship quality with prosocial behavior among Chinese adolescents. This study will have both theoretical and practical significance. Theoretically, it enriches the understanding of PYD mechanisms by exploring them from cognitive and motivational perspectives. Practically speaking, this aligns with the goals of China’s ideological and political education, which emphasize cultivating inspiration and a collectivist spirit, offering insights into educational practices that promote prosocial behavior among Chinese youth.

### 1.1 Relationship quality and prosocial behavior

Familial, school, and peer relationships form the primary social contexts for adolescents. Research consistently indicates that the quality of these relationships is a positive predictor of adolescents’ prosocial behavior. For instance, Li et al. (26) suggested that positive parent–child attachment fosters prosocial behavior in children and adolescents. Longobardi et al. (27) found that the teacher–student relationship was positively associated with students’ prosocial behavior. Zhou et al. (28) revealed bidirectional relationships between positive friendship quality and prosocial behavior. Therefore, in the present study, we hypothesize that higher-quality parent–child relationships, teacher–student relationships, and friendships at T1 will be positively associate with higher levels of prosocial behavior at T3 (H1).

H1a: The quality of parent–child relationships positively will associate with adolescents’ prosocial behavior.

H1b: The quality of teacher–student relationships positively associate with adolescents’ prosocial behavior.

H1c: The quality of friendships positively associates with adolescents’ prosocial behavior.

### 1.2 Relationship quality and meaning in life

Meaning in life encompasses an individual’s comprehensive understanding of themselves, their environment, their role in the world, and their life’s purpose, goals, and mission (29–32). A central feature of meaning in life is its broad perspective, incorporating considerations that extend beyond the self (20). Meaning in life comprises two dimensions: the presence of meaning, reflecting a sense of coherence, purpose, and significance in life, and the search for meaning, representing an active effort seeking to understand and enhance life’s purpose and significance (33, 34). Social support and connection play a crucial and bidirectional role in shaping meaning in life (25, 35, 36). Research indicates that socially grounded feelings, such as a sense of belonging (24) or connectedness (37), significantly enhance meaning in life. Daily diary studies reveal that individuals perceive days with positive social events as the most meaningful (38). Furthermore, the quality of social connections is strongly associated with aspects of meaning in life, with individuals reporting a greater sense of purpose on days with better social interactions (39) and in relationships characterized by higher support and lower strain (40). Conversely, experiences of social exclusion (41) or interpersonal rejection (42) are linked to diminished perceptions of life’s meaning. Among the important relationships, familial relationships have been consistently identified as the most significant contributors to meaning in life (43, 44). However, there is a relative scarcity of studies examining the associations between teacher–student relationships and meaning in life (45), as well as between friendships and meaning in life (46).

In the present study, we hypothesize that the quality of parent–child relationships, teacher–student relationships, and friendships positively influences adolescents’ presence of meaning in life (H2) and search for meaning in life (H3). Specifically, supportive and nurturing relationships within these primary social contexts are expected to be associated with adolescents’ greater sense of purpose and significance in life and their motivation to actively explore life’s deeper significance. High-quality parent–child relationships, characterized by warmth, trust, and emotional support, provide a secure foundation for adolescents to develop a coherent and purposeful understanding of their lives. These relationships also foster an environment where adolescents feel encouraged to explore and reflect on their personal goals and values, driving their search for meaning. Similarly, positive teacher–student relationships, marked by encouragement, mutual respect, and constructive feedback, not only contribute to academic and personal growth but also help adolescents perceive their lives as meaningful and purposeful. Teachers, as role models and mentors, can inspire students to seek answers to life’s broader questions and cultivate a deeper understanding of their roles in society. Friendships, a vital source of emotional support and social learning during adolescence, play a key role in fostering feelings of belonging and connectedness, which are closely associated with a sense of life meaning. Additionally, engaging in meaningful conversations and shared experiences with friends can stimulate adolescents’ curiosity and motivate their search for life’s purpose. In summary, we propose that the quality of these three key relationships positively associate with both the presence and pursuit of meaning in life.

H2a: The quality of parent–child relationships positively associate with adolescents’ presence of meaning in life.

H2b: The quality of teacher–student relationships positively associate with adolescents’ presence of meaning in life.

H2c: The quality of friendships positively adolescents’ presence of meaning in life.

H3a: The quality of parent–child relationships positively associate with adolescents’ search for meaning in life.

H3b: The quality of teacher–student relationships positively associate with adolescents’ search for meaning in life.

H3c: The quality of friendships positively associates with adolescents’ search for meaning in life.

### 1.3 Meaning in life and prosocial behavior

Previous studies suggest that the two dimensions of meaning in life may relate differently to prosocial behavior. Evidence for the presence of meaning is relatively consistent, with longitudinal studies showing positive associations with subsequent prosocial behavior (20, 23). A clear sense of purpose and significance may provide direction for behavior and support actions that are consistent with personal values. Thus, adolescents with a stronger presence of meaning may be more likely to engage in prosocial behavior.

In contrast, the role of the search for meaning is more complex. Searching for meaning may reflect constructive exploration of life goals and values, but it may also arise from uncertainty or a perceived lack of meaning and be accompanied by psychological distress (33, 47, 48). Xie et al. (23) found no longitudinal association between the search for meaning and prosocial behavior, and its implications may vary with the presence of meaning and stressful life events (47). Given this mixed evidence, we examine the association between the search for meaning and prosocial behavior.

H4: Adolescents’ presence of meaning in life positively associates with their prosocial behavior.

H5: Adolescents’ search for meaning in life is associated with prosocial behavior.

### 1.4 The present study

**Figure 1:**
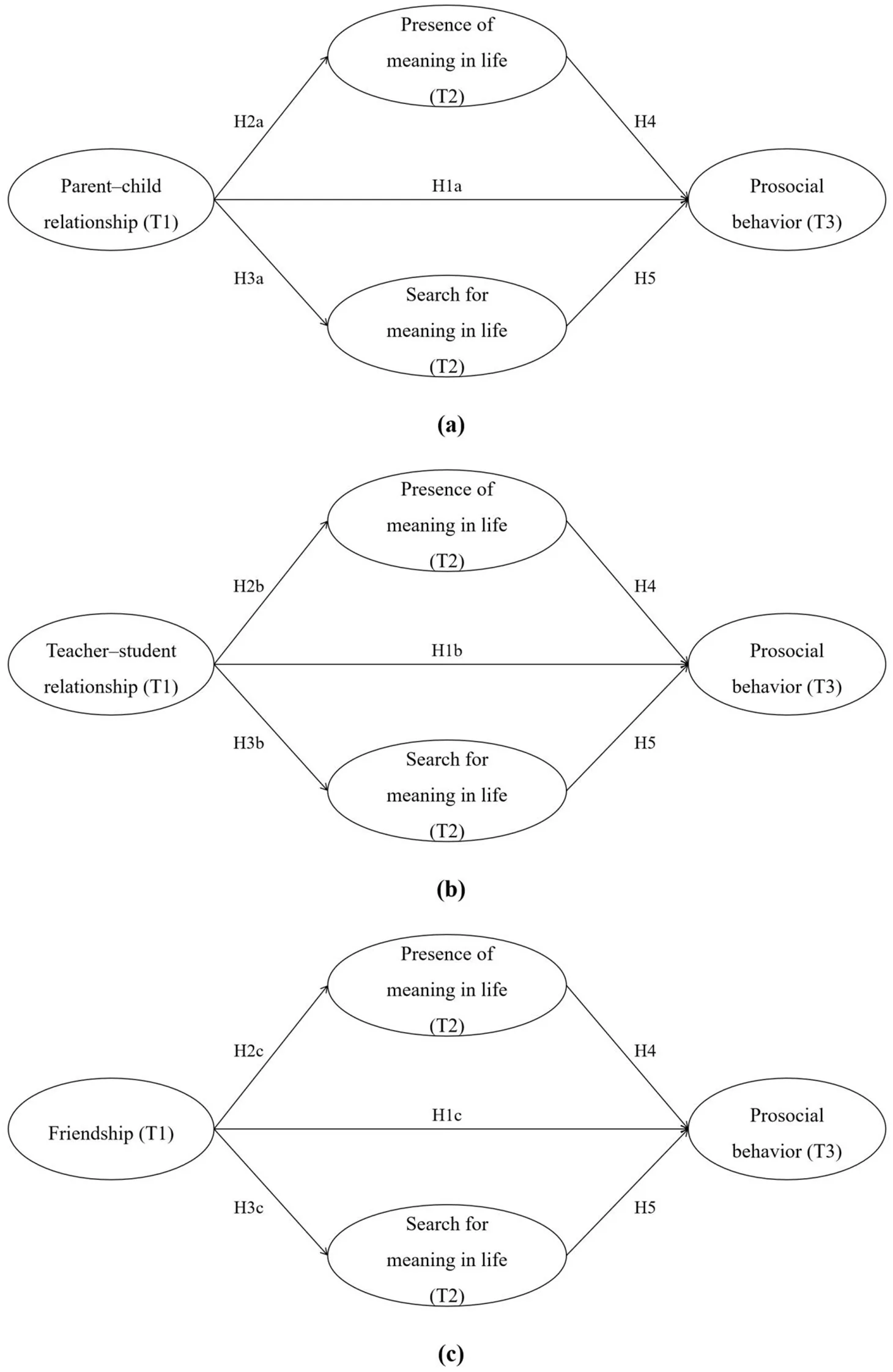
The proposed research model. **(a)** Hypothesized mediation model for parent_child relationship. **(b)** Hypothesized mediation model for teacher_student relationship. **(c)** Hypothesized mediation model for friendship.

In this study, we aim to examine the prospective associations between parent–child relationships, teacher–student relationships, and friendships on adolescents’ prosocial behavior, with a particular focus on the mediating roles of the presence of meaning in life and the search for meaning in life. To establish a temporal ordering among the focal constructs, we adopted a three-wave longitudinal research design, with the independent variables, mediators, and outcome variables measured at three successive time points, each six months apart. The research model is summarized in **Figure 1**.

## 2 Methods

### 2.1 Participants and procedures

The participants of this study were students from a junior high school in Hunan Province, selected through cluster sampling by class. Informed consent was obtained from the school, students, and their parents. The first measurement (T1) was conducted in May 2021 to assess parent–child relationships, teacher–student relationships, and friendships. The second measurement (T2) took place in November 2021, focusing on the presence of meaning in life and the search for meaning in life. The third measurement (T3) was conducted in May 2022 to evaluate prosocial behavior. Data were collected using paper-and-pencil questionnaires at all three assessment periods. Although the number of valid responses varied slightly across the three waves due to absences and student transfers, the overall retention rate was high. After screening and matching the questionnaires from all three measurements, a total of 671 valid responses were included in the final analysis. Compared with the valid T1 sample, 18 participants were not retained in the matched longitudinal sample, resulting in a retention rate of 97.4% and an attrition rate of 2.6%. Attrition analyses revealed no significant differences between retained and unmatched participants in baseline demographic characteristics and study variables (*ps* >.05), suggesting that attrition was unlikely to introduce substantial bias into the longitudinal. There was no structured participant attrition across the surveys. After a rigorous evaluation and merging of the questionnaires from all three measurements, a total of 671 valid responses were included in the final analysis. Among them, 347 were male students (51.7%) and 324 were female students (48.2%). At T2, the participants’ ages ranged from 11 to 15 years, with a mean age of 13.33 years (*SD* = 0.63).

### 2.2 Measures

#### 2.2.1 Parent–child relationship

The Parent–Child Closeness Scale, originally developed by Buchanan et al. (49) and revised by Zhang et al. (50), was used to assess the relationships between adolescents and their fathers and mothers separately. The scale consists of 9 items for father–child relationships and 9 items for mother–child relationships, rated on a 5-point Likert scale ranging from 1 (“*not at all true*”) to 5 (“*very true*”). Higher scores indicate closer parent-child relationships. For statistical analysis, the mean score of all 18 items (Cronbach’s alpha = 0.948) was calculated as the measure of parent–child relationships.

#### 2.2.2 Teacher–student relationship

The Teacher–Student Relationship Subscale from the My Class Questionnaire, developed by Jiang (51), was used to measure students’ perceptions of the relationships with their head teachers. The subscale includes 8 items rated on a 5-point Likert scale, where 1 indicates “*never*” and 5 indicates “*always*”. Higher mean scores reflect better teacher–student relationships in this study, with a Cronbach’s alpha value of 0.952.

#### 2.2.3 Friendship

This study employed the Friendship Quality Questionnaire–Short Form, revised by Zhou et al. (52), to measure friendship quality among children and adolescents. The questionnaire assesses six dimensions of friendship quality: (1) affirmation and care, (2) help and guidance, (3) companionship and recreation, (4) intimacy and communication, (5) conflict resolution strategies, and (6) conflict and betrayal. The first five dimensions capture the positive aspects of friendship quality, while the sixth dimension reflects its negative aspects. In line with prior research (52), the mean score of the items from the first five dimensions (Cronbach’s alpha = 0.908) was used to represent positive friendship quality in this study, with higher scores indicating better friendship quality; the sixth dimension was excluded from the analyses.

#### 2.2.4 Meaning in life

The Chinese Meaning in Life Questionnaire, originally developed by Steger et al. (34) and validated by Wang and Dai (53), was used in this study. The questionnaire employs a 7-point Likert scale, ranging from 1 (“*strongly disagree*”) to 7 (“*strongly agree*”). The presence of meaning in life and the search for meaning in life were each measured using 5 items (Cronbach’s alpha = 0.767 and 0.911, respectively), with higher mean scores indicating higher levels of the respective variables.

#### 2.2.5 Prosocial behavior

The Prosocial Tendencies Measure, revised by Kou et al. (54), was used in this study. The scale consists of 26 items covering six dimensions: public, anonymous, altruistic, compliant, emotional, and dire prosocial tendencies. Participants rated each item on a 5-point Likert scale, where 1 indicates “*not at all like me*” and 5 indicates “*very much like me*”. In this study, the mean score of all 26 items (Cronbach’s alpha = 0.973) was calculated as the measure of prosocial behavior, with higher scores indicating greater prosocial tendencies.

### 2.3 Data analysis

Data analysis, including descriptive statistics and mediation analyses, was conducted using Statistical Product and Service Solutions Automatically (SPSSAU, https://spssau.com/indexs.html) in this study. Relying on its standard algorithms and automated output. While it has limitations, such as in highly customized programming, these do not affect the analyses in this study, as conventional mediation modeling via PROCESS Model 4 was employed.

## 3 Results

### 3.1 Common method bias test

To assess potential common method bias, Harman’s single-factor test was conducted (6). Thirteen factors had eigenvalues greater than 1, with the first factor accounting for 24.83% of the variance. The variance explained by the first factor was below the 40% threshold, suggesting that common method bias was not a serious concern in this study.

### 3.2 Descriptive statistics

The means, standard deviations, and Pearson correlation coefficients of the variables are presented in **Table 1**.

**Table 1:** Descriptive statistics.

Variable	*M*	*SD*	1	2	3	4	5	6
1. Parent–child relationship (T1)	3.429	0.905	1					
2. Teacher–student relationship (T1)	4.295	0.897	0.228^**^	1				
3. Friendship (T1)	3.537	0.851	0.243^**^	0.176^**^	1			
4. Presence of meaning in life (T2)	4.662	1.329	0.224^**^	0.140^**^	0.205^**^	1		
5. Search for meaning in life (T2)	5.306	1.477	0.148^**^	0.043	0.207^**^	0.622^**^	1	
6. Prosocial Behavior (T3)	3.746	0.737	0.144^**^	0.125^**^	0.158^**^	0.156^**^	0.088^*^	1

^*^ *p* < 0.05, ^**^p < 0.01.

### 3.3 Mediation analyses

**Figure 2:**
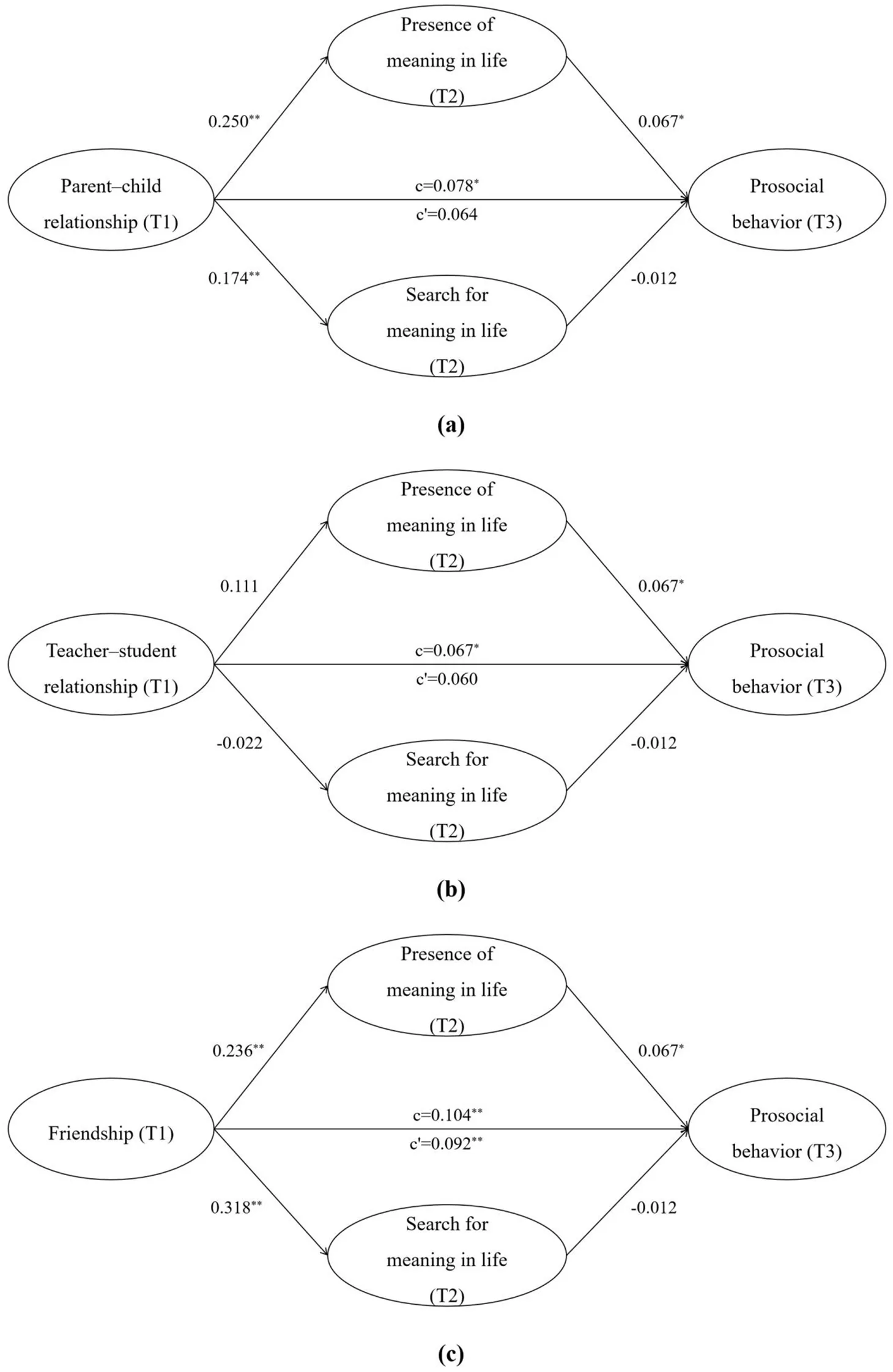
Results of the longitudinal mediation analysis. **(a)** Mediation results for parent_child relationship. **(b)** Mediation results for teacher_student relationship. **(c)** Mediation results for friendship. *P < 0.05, **p < 0.01.

As shown in **Figure 2**, the results of the longitudinal mediation analysis revealed that the total effects of relationship quality on prosocial behavior were significant across all three models. Specifically, parent–child relationships at T1 positively predicted prosocial behavior at T3, and the same pattern was observed for teacher–student relationships and friendships, thereby supporting H1. Among the three types of relationships, friendships demonstrated the strongest total effect on prosocial behavior.

In terms of mediation effects, the results showed that the pathway from relationship quality to prosocial behavior through the presence of meaning in life was significant for parent–child relationships and friendships. Specifically, parent–child relationships at T1 significantly predicted the presence of meaning in life at T2, which in turn positively predicted prosocial behavior at T3. Similarly, friendships at T1 were positively associated with the presence of meaning in life at T2, which mediated their effect on prosocial behavior at T3. In contrast, teacher–student relationships at T1 did not significantly predict the presence of meaning in life at T2, indicating that the mediating role of the presence of meaning in life was not significant in the teacher–student relationship model. These findings supported H2a, H2c, and H4 but not H2b.

However, unlike the presence of meaning in life, the search for meaning in life did not exhibit significant mediation effects in any of the three models. Although both parent–child relationships and friendships at T1 significantly predicted the search for meaning in life at T2, the search for meaning in life did not significantly predict prosocial behavior at T3, supporting H3a and H3c but not H5. The search for meaning in life was not significantly associated with prosocial behavior at T3. Thus, H3a and H3c were supported, whereas the exploratory association proposed in H5 was not significant. Additionally, teacher–student relationships at T1 did not significantly predict the search for meaning in life at T2, thereby rejecting H3b.

When controlling for the mediators, the direct effects of relationship quality on prosocial behavior varied across the three models. For the parent–child relationship model, the direct effect on prosocial behavior was nonsignificant, indicating a full mediation effect through the presence of meaning in life. In contrast, for the friendship model, the direct effect on prosocial behavior remained significant, suggesting a partial mediation effect, with the indirect effect accounting for 15.34% of the total effect.

In summary, the findings highlight the distinct roles of various relationships and different dimensions of meaning in life in shaping adolescents’ prosocial behavior.

## 4 Discussion

### 4.1 Primary findings and implications

The primary findings of this study reveal that the total effects of relationship quality on prosocial behavior were significant for all three types of relationships, with friendships showing the strongest effect. The presence of meaning in life mediated the effects of parent–child relationships and friendships on prosocial behavior, with a full mediation observed in the parent–child relationship model and a partial mediation in the friendship model. However, no significant mediation effects were found for the search for meaning in life in any of the models. Teacher–student relationships did not significantly predict either type of meaning in life, and their influence on prosocial behavior was weaker compared to parent–child relationships and friendships. These results underscore the differential roles of various relationships and dimensions of meaning in life in shaping adolescents’ prosocial behavior.

The effects of relationship quality on adolescents’ prosocial behavior found in the current study align with prior research, such as Li et al. (26), Longobardi et al. (27), and Zhou et al. (28). However, this study might be the first to directly compare the effects of different types of relationships on adolescents’ prosocial behavior. The finding that friendships exerted the strongest effect highlights the critical role of peer relationships during adolescence. Adolescence is a developmental stage marked by increased reliance on peers as sources of emotional support, social learning, and identity formation. Strong, high-quality friendships can foster a sense of belonging and connectedness, which are crucial for promoting prosocial behavior (55–57). This suggests that interventions aimed at enhancing adolescents’ social development should focus on cultivating healthy and supportive peer relationships. From an ecological systems perspective (58), these efforts can be extended across multiple relational contexts. At the peer level, schools can implement structured peer mentoring and cooperative learning to systematically strengthen friendships. At the family level, parents should create supportive environments and engage children in shared community activities to nurture a sense of social contribution. At the teacher level, training programs may incorporate affective communication modules to help educators balance their authoritative role with emotional support. These strategies, working in concert across proximal and distal contexts, may reinforce adolescents’ prosocial development in a more sustained and coherent manner. For example, schools could implement peer mentoring programs or cooperative learning activities to strengthen friendships and encourage positive social interactions. Additionally, parents and educators should be aware of the growing importance of peer relationships during adolescence and create environments that support the development of strong, prosocial peer bonds. These efforts can help adolescents build the social skills and empathy needed to engage in prosocial behaviors, ultimately contributing to their overall well-being and positive social development.

The significant mediating role of the presence of meaning in life, while not directly studied in prior research, is consistent with existing findings, such as those of Zhang et al. (20) and Xie et al. (23), and underscores its importance as a psychological mechanism through which high-quality relationships promote prosocial behavior in adolescents. These findings reveal that the mechanisms of PYD extend beyond social-emotional learning to include cognitive and motivational perspectives. This is particularly significant in the context of Chinese education, which places a strong emphasis on ideological and moral development. Rooted in Confucian traditions, Chinese education has long promoted life purposes centered on societal contribution, encapsulated in the ideals of “cultivating oneself, managing one’s family, governing the state, and bringing peace to the world” (59–61). This Confucian vision finds its contemporary expression in China’s core socialist values, notably “friendship”, which explicitly promotes mutual care and helping behavior as civic virtues (62). The findings of this study provide empirical support for these efforts by demonstrating that high-quality relationships play a key role in fostering a sense of purpose oriented toward prosocial contributions. This highlights the potential for expanding the scope of moral education in China to focus on nurturing meaningful relationships that inspire adolescents to see societal contribution as a central element of their life purpose. By doing so, these findings offer a broader, evidence-based pathway for strengthening moral education and cultivating prosocial behavior among Chinese youth.

Importantly, the mediation patterns differed across relationship contexts, suggesting that meaning in life represents an important but not exclusive pathway linking adolescents’ social relationships with prosocial behavior. The full mediation observed in the parent–child relationship model indicates that the association between supportive family relationships and prosocial behavior may be primarily explained by adolescents’ internalization of meaning and values derived from family experiences. Parents may foster adolescents’ prosocial orientations through emotional security, value transmission, and moral socialization processes (63).

In contrast, the partial mediation found in the friendship model suggests that friendships may contribute to adolescents’ prosocial behavior through additional peer-related mechanisms beyond meaning construction. During adolescence, friendships provide important contexts for social learning, behavioral modeling, and the development of peer norms. Recent empirical studies have shown that adolescents’ prosocial behaviors can be shaped by peer environments through processes of peer modeling and normative influence (64–66). For example, adolescents tend to adjust their behavioral norms according to the prosocial norms expressed by peers, particularly within meaningful social networks (65), and positive peer contexts can facilitate the development of prosocial behavior over time (64). Therefore, the remaining direct association between friendship quality and prosocial behavior may reflect the unique role of peers as immediate socialization agents who provide opportunities for observing, practicing, and reinforcing prosocial behaviors. These mediation patterns should be interpreted as statistical associations and do not, by themselves, establish causal mechanisms (67).

### 4.2 The nonsignificant context of teacher–student relationship

The nonsignificant effect of teacher–student relationships on the presence and search for meaning in life may stem from several contextual and developmental factors.

First, the emotional depth of teacher–student relationships is often more limited compared to parent–child relationships and friendships. While teachers play a crucial role in academic guidance and behavioral support, their interactions with students may lack the intimacy and personal investment needed to foster a strong sense of belonging or connectedness (68). The formal and structured nature of these relationships may constrain their influence on adolescents’ internalization of life’s meaning.

Second, cultural expectations and role perceptions rooted in the Confucian tradition within Chinese educational settings may further contribute to the nonsignificant findings (59, 61). In Chinese cultural contexts, teacher–student relationships are shaped by both relational closeness and hierarchical expectations. The Confucian tradition of respecting teachers and valuing education emphasizes teachers’ roles not only as transmitters of knowledge but also as moral guides, as reflected in the classical educational text Xueji (69, 70). In Confucian-heritage cultures, teacher–student relationships have historically been characterized by hierarchical expectations, with teachers occupying positions of authority and moral guidance (71). This cultural legacy continues to influence contemporary understandings of teachers’ professional roles in Chinese societies, where teachers are often positioned as both educators and moral exemplars (72). At the same time, recent research suggests that respect for teachers in Confucian cultural contexts is multidimensional, involving both positive appreciation and authority-related orientations, which may have different implications for students’ perceptions of teacher–student relationships (73). Although Confucian educational traditions emphasize meaningful teacher–student relationships and moral cultivation (74), the coexistence of teacher authority and relational closeness may shape the extent to which adolescents perceive teachers as sources of emotional support and personal meaning. In many Chinese schools, teacher–student relationships are often characterized by a focus on discipline, authority, and academic achievement rather than emotional connectedness (60). Adolescents may primarily view teachers as authority figures rather than sources of emotional support, which could weaken the potential impact of these relationships on their broader developmental outcomes.

Finally, the nonsignificant findings may also be attributed to the variability and complexity of teacher–student relationship quality. One important factor is the continuity of teacher–student relationships; for instance, whether teachers continue to instruct the same students as they progress to higher grades. Disruptions in these relationships could weaken their influence on adolescents’ personal and social development. Additionally, students are often influenced by multiple teachers, not just their head teacher. Given differences in teachers’ personalities and teaching styles, some students may form stronger emotional connections with certain subject teachers rather than their head teacher. However, the measures used in this study focused solely on the relationship with the head teacher, which may not have been comprehensive enough to capture the broader and more nuanced effects of teacher–student relationships on the outcomes.

### 4.3 The nonsignificant mediator of search for meaning in life

The nonsignificant mediating role of the search for meaning in life observed in this study is consistent with prior research that underscores the complex and often inconclusive effects of this construct. While the presence of meaning in life has been strongly associated with positive outcomes such as well-being, life satisfaction, and resilience (e.g., 32, 75), the search for meaning operates in a more nuanced manner, serving as both a stressor and a facilitator of developmental outcomes (33, 47, 48).

On the one hand, the search for meaning can reflect unmet needs and an absence of life purpose, often linked to maladjustment and psychological distress (30, 76, 77). For individuals actively searching for meaning, the process may be accompanied by heightened existential anxiety or distress, as it highlights the gap between their current state and an idealized sense of purpose or coherence (78). Adolescents, in particular, may struggle with this search if they lack supportive resources or a clear framework to construct meaning from their experiences.

On the other hand, the search for meaning can serve as a powerful growth-oriented mechanism, allowing individuals to reinterpret negative experiences, integrate challenging perspectives, and overcome adversity (79, 80). This constructive aspect of the search for meaning may facilitate long-term well-being and personal growth, as individuals adapt to challenges and redefine their goals and values in the face of adversity. This dual nature of the search for meaning underscores its complexity, as it can lead to either adaptive or maladaptive outcomes depending on individual and contextual factors.

In the present study, the nonsignificant findings may also be partially explained by the developmental stage of the participants. The sample consisted of early adolescents, who are generally in the exploratory phase of constructing meaning. During this phase, the search for meaning is often less goal-directed and more experimental, reflecting the process of identity formation and self-discovery rather than yielding immediate, measurable behavioral outcomes such as prosocial actions. Unlike the presence of meaning, which provides a coherent framework for guiding behavior, the exploratory nature of the search for meaning may have less direct influence on prosocial tendencies at this stage.

### 4.4 Limitations and future directions

This study has several limitations that should be noted. First, the sample was drawn from a single region in China, which may limit the generalizability of the findings to other cultural or regional contexts; future research should include more diverse populations. Second, the focus on head teacher–student relationships excluded the potential influence of relationships with other teachers, like subject teachers and counselors, which could be explored in future studies. Third, the six-month intervals between measurements may not fully capture the dynamic nature of these relationships; more frequent measurements could provide greater insight. Fourth, potential moderators such as adolescents’ self-construal, which might influence the nonsignificant findings regarding the search for meaning in life, should be considered in future research. Lastly, reliance on self-report measures introduces potential biases; future studies should incorporate multi-informant or observational methods. Fifth, all study variables were assessed using adolescent self-reports. This single-informant design may introduce common method variance and social desirability bias and does not capture the perspectives of parents, teachers, or peers. Future studies should incorporate multi-informant or observational assessments. By addressing these limitations, future research can provide a more comprehensive understanding of the factors that shape adolescents’ prosocial development.

## 5 Conclusions

In this study, we explored how parent–child relationships, teacher–student relationships, and friendships associate with adolescents’ prosocial behavior through the mediating roles of meaning in life. Using a longitudinal mediation model, the results showed that the quality of parent–child relationships and friendships at T1 positively predicted both the presence and search for meaning in life at T2, but only the presence of meaning in life at T2 positively predicted prosocial behavior at T3. These findings not only deepen our understanding of the PYD mechanism by highlighting the cognitive and motivational pathways through which relationships foster prosocial behavior, but also offer valuable insights for enhancing the moral education framework in China, emphasizing the role of meaningful relationships in shaping adolescents’ sense of purpose and societal contribution.

## Data Availability

The original contributions presented in the study are included in the article/supplementary material. Further inquiries can be directed to the corresponding authors.
